# The impact of oxidative stress, inflammation, and senescence on the maintenance of immunological memory in the bone marrow in old age

**DOI:** 10.1042/BSR20190371

**Published:** 2019-05-14

**Authors:** Erin Naismith, Luca Pangrazzi

**Affiliations:** Department of Immunology, Institute for Biomedical Aging Research, University of Innsbruck, Rennweg 10, Innsbruck, Austria

**Keywords:** aging, bone marrow, immunosenescence, immunological memory, inflammation, ROS

## Abstract

The bone marrow (BM) provides a preferential survival environment for the long-term maintenance of antigen-experienced adaptive immune cells. After the contact with antigens, effector/memory T cells and plasma cell precursors migrate to the BM, in which they can survive within survival niches in an antigen-independent manner. Despite this, the phenotype of adaptive immune cells changes with aging, and BM niches themselves are affected, leading to impaired long-term maintenance of immunological memory in the elderly as a result. Oxidative stress, age-related inflammation (inflammaging), and cellular senescence appear to play a major role in this process. This review will summarize the age-related changes in T and B cell phenotype, and in the BM niches, discussing the possibility that the accumulation of highly differentiated, senescent-like T cells in the BM during aging may cause inflammation in the BM and promote oxidative stress and senescence. In addition, senescent-like T cells may compete for space with other immune cells within the marrow, partially excluding effector/memory T cells and long-lived plasma cells from the niches.

## Introduction

The bone marrow (BM) is renowned for hematopoiesis, the formation of the cellular components that comprise our blood, but it has also been described as the preferred site for the maintenance and survival of long-living immune cells [1,2]. Over time, the immune system undergoes age-related changes, which are often referred to as immunosenescence [3]. In particular, a drop in cellularity can be observed in the BM, while the remaining space is simultaneously occupied by fat [4]. How this change in BM composition affects the maintenance of adaptive immune cells is continuously being explored. The BM is made up of parenchymal hematopoietic cells and mesenchymal support cells [5,6]. The reticular stromal cells form distinct niches, which provide survival factors to long-living immune cells which reside in the BM and can persist for many years [2]. The ways in which immunosenescence effects the BM niches and the BM environment, the coetaneous changes in B and T cells in the BM, as well as the impact of cytomegalovirus (CMV), a known driver of immunosenescence, will be discussed in this review.

## Long-term maintenance of adaptive immune cells in the BM

### Plasma cells

A key role in memory T cell homing and survival has been attributed to the BM. Resting memory T cells specific for systemic pathogens such as tetanus, rubella, mumps, and measles can be found in the human BM [7]. After antigens have been cleared, some of the newly generated adaptive cells are maintained for long periods of time in the BM, where a high amount of T cell and plasma cell survival factors are produced [8]. The maintenance of immunological memory is mediated by cytokine/chemokine-producing cells, such as stromal cells and eosinophils [9]. Specific areas in the BM that are rich in these molecules, known as niches, are believed to support the homeostatic proliferation of plasma cells and effector/memory T cells. Several cell types contribute to the organization of the plasma cell niche in the mouse BM. Plasmablasts generated in germinal centres migrate to the BM after interacting with the chemokine CXCL-12, predominantly expressed by CXCL-12-abundant reticular stromal cells, where they mature into plasma cells [9,10]. The majority of plasma cells show stable contact with reticular stromal cells and are sessile in their position [11,12]. The survival of plasma cells in the BM is mainly supported by two cytokines, a proliferation-inducing ligand (APRIL), and IL-6, produced by cells of hematopoietic origin such as granulocytes; in particular eosinophils, megakaryocytes, and monocytes [12–14]. Neutralization of both APRIL and IL-6 has shown to deplete antigen-specific plasma cells in the BM [13]. Long-lived plasma cells can be maintained in the niches for decades, and although they may not proliferate they are highly active in term of protein synthesis [8,15]. Interestingly, it has recently been suggested that the classical B cell marker CD19, expression of which increases during B cell development, may be produced by short-lived, but not on long-lived human plasma cells (LLPCs) [15]. Thus, the phenotype of LLPCs may somehow be similar to immature B cells. Despite this, LLPCs show unique features, such as a distinct antibody repertoire and RNA transcriptome [15].

### Effector/memory T cells

**Figure 1:**
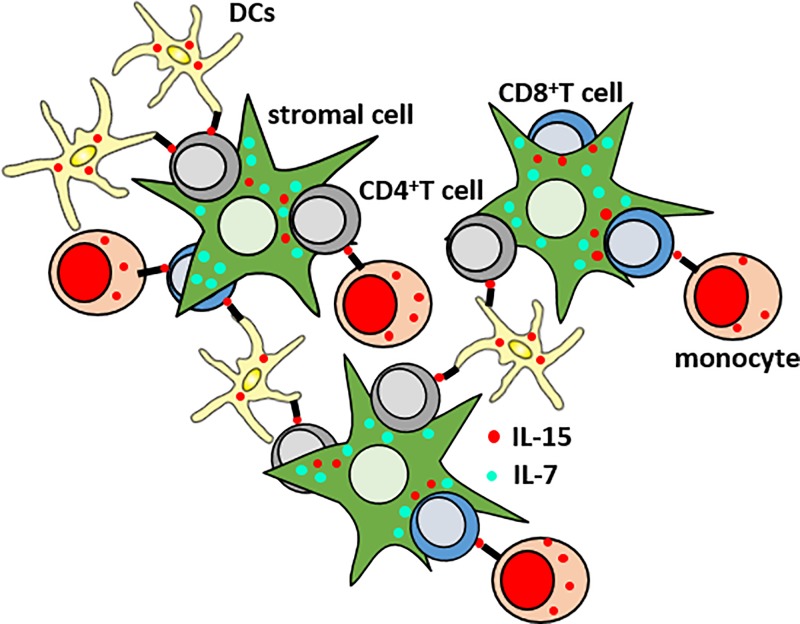
Hypothetical structure of a BM niche for CD8^+^ and CD4^+^ effector/memory T cells Myeloid cells (monocytes and DCs) and stromal cells produce and trans-present IL-15 to effector/memory CD8^+^ and CD4^+^ T cells. IL-7 is produced and secreted mostly by stromal cells.

The BM has additionally been described as a preferred site for the survival of effector/memory CD4^+^ and CD8^+^ T cells [16–18]. In mice, a major proportion of CD4^+^ memory T helper cells migrate to the BM within 3–8 weeks after their generation, and are maintained there for a long time in distinct survival niches [19]. Memory CD4^+^ T cells are maintained in niches organized by IL-7^+^ stromal cells, which express vascular cell adhesion molecule 1 (VCAM-1) and collagen XI [20]; [19]. IL-7 is known to promote the transition of CD4^+^ T cells from ‘short-lived’ to ‘long-lived’, and represents the main survival factor for CD4^+^ memory T cells [21]. In the BM, CD69 expression on CD4^+^ memory T cell precursors is necessary for the rolling of these cells on BM sinusoids, which represents the first step these cells take before entering the BM matrix. After this initial weak adhesion, the trans-migration of effector cells into the BM through the sinusoidal endothelia is possible thanks to CD49b (integrin α2). CD49b guides the cell migration in the direction of the niches via binding to collagen II and finally to collagen XI, expressed by IL-7^+^ stromal cells. Once memory CD4^+^ T cells reach their final position in the niche, they can be maintained there for long periods. In addition to IL-7, the cytokine IL-15 is required for the survival of CD4^+^ memory T cells [22], and interactions between CD4^+^ T cells and IL-15^+^ cells in the BM have previously been described [23]. The maintenance of effector/memory CD8^+^ T cells requires both IL-7 and IL-15 [24]. While a CD4^+^ memory T cell niche has been described in mice, the structure of a memory CD8^+^ T cell niche is not yet known, in neither mice nor in humans. IL-7 produced by BM stromal cells binds to IL-7Rα expressed on CD8^+^ effector/memory T cells. Myeloid and stromal cells produce and trans-present IL-15, not only to memory CD8^+^ T cells but also to effector/highly differentiated CD8^+^ T cells, which express high levels of the IL-2/IL-15Rβ chain (CD122) and γ chains (CD132) [25–27]. A schematic representation of a hypothetical BM niche for CD8^+^ and CD4^+^ T cells is shown in Figure 1. In memory CD8^+^ T cells, cytokines IL-7 and IL-15 synergize. While IL-15 is mainly a T cell proliferation factor, the generation and survival of memory T cells depend on IL7Rα signaling [28]. Indeed, IL7Rα^+^KLRG-1^−^ CD8^+^ memory progenitor effector T cells (MPEC) are preferentially supported by IL-7, while IL7Rα^−^KLRG-1^+^ CD8^+^ ‘short living’ effector cells (SLEC), which also include highly differentiated CD8^+^ T cells, are maintained by IL-15 [29]. Whether MPEC and SLEC are also present in the BM has not yet been investigated. Despite this, it is now clear that survival and proliferation of effector/memory CD8^+^ T cells is effective, only when both IL-7 and IL-15 are produced. IL-15 is present in high concentrations and has a high bioavailability in the BM [23,30,31]. IL-15-expressing cells can be found throughout the entire organ, particularly around small blood vessels [32]. The highest IL-15 expression was found in VCAM-1^+^ reticular stromal cells, which also express IL-7. IL-15 is also produced by various myeloid cell types, such as dendritic cells (DCs), monocytes, and macrophages [33–35].

Two major theories exist on the long-term maintenance of memory cells in the BM: the quiescent niche and the self-renewal niche [36,37]. Evidence of resident populations in the BM, based on exclusive specificity repertoires, supports the quiescence niche theory, which suggests that memory T cells specific for several viral pathogens typically encountered during childhood permanently inhabit and are exclusively maintained in the BM [6]. Additionally, murine studies have shown that antigen-experienced CD4^+^ T cells relocate to the BM during the contraction phase of a reaction, and after 120 days, antigen experienced cells were only detectable in the BM [19]. Alternatively, supporting the self-renewal niche theory, evidence also exists that memory T cells home to the BM and persist there over time, in constant exchange with the peripheral blood (PB) T-cell pool [38]. The *in situ* labeling of sheep BM cells, which subsequently reached the spleen and other secondary lymphoid organs, supports the theory that the BM is a temporary stopping point for recirculating memory T cells, which find in the BM a proper environment to be maintained by homeostatic proliferation [36,37,39]. Additionally, parabiosis experiments between CD45-congenic mice showed that comparable numbers of CD45.1^+^ and CD45.2^+^ antigen specific memory CD8^+^ T cells were detectable in the BM of each mouse [40]. In addition to these two theories, a third theory suggests that both niches may exist [37]. The two-niche theory bridges the two existing ideas, suggesting that recirculating memory T cells are maintained within two distinct niches in the BM, one quiescent or resting niche, and one self-renewing or recirculating niche [37]. According to the two-niche theory, only when both niches are functioning properly, an effective secondary response to antigen can occur. A defective self-renewal niche could explain a gradual drop in T cell numbers during the memory phase, while a defect in the quiescent niche could lead to improper expansion upon re-exposure to antigens [37]. The debate on how life-long memory T cells persist in the BM is ongoing, with each different theory providing useful perspectives on certain pathologies.

## Age-related changes of adaptive immune cells in the BM

The age-related decline in the functionality of the immune system, which leads to impaired immune responses in the elderly, is known as immunosenescence [41,42]. This process affects both branches of the adaptive immunity and brings to reduced responses to infections and vaccinations in old age [42].

### B cells

Once the immune system has encountered a pathogen, it can develop immunological memory, allowing a faster and more effective response should a second exposure occur [43]. B lymphocytes are key players in the formation and maintenance of protective immunity, and understanding how age impacts the B-cell lineage is fundamental to understanding immunosenescence [44]. Early descriptive mouse studies show reduced numbers, as well as reduced functionality, of B cell precursors in the BM with aging [45]. A decline has also been reported in the size and number of germinal centers (GCs), immunological sites with the lymph nodes and spleen where mature B cells proliferate, differentiate, hyper-mutate, and undergo class switching, resulting in the generation of antibodies with lower avidity and affinity [46].

Cancro *et al.* reported changes with age within the different B cell subsets, as well as shifts in the diversity and clonotypic composition of the antigen recognition repertoire [44]. The authors also noted a decrease in B cell progenitors, reduced BM progenitor output, and intrinsic changes within the hematopoietic stem cells (HSCs), as well as repertoire shifts and reduced BCR diversity in the peripheral pre-immune B cells [44]. A decrease in B cell progenitors, reduced BM progenitor output, intrinsic changes within the HSCs, as well as repertoire shifts and reduced BCR diversity in the peripheral pre-immune B cells have additionally been observed [47]. Within the B cell repertoire, a decline in both the percentage and number of switched memory B cells was seen in aged mice, while IgM memory B cells decreased in number but not percentage. Naive B cells increased in percentage; however, the absolute number was not significantly different [48]. B cells with immune regulatory properties have been identified in mice, and they are reported to regulate immune function via the production of IL-10 [49]. The regulatory function of these cells was observed to be impaired with age, and both the percentage and number of regulatory B cells in the PB decreased during aging. The impairments in regulatory B cell functionality has been identified as a contributing factor towards increased risk of RA in elderly [49]. A recent study from the same group; however, has indicated that physical activity and exercise had a large positive impact on this reduction [50]. Poor response to vaccination, a higher rate of new infections, poor memory responses, and an increased rate of auto-immune disorders and increased tumor incidence observed in the elderly indicate that antigen experienced B cells are also impacted during aging [51,52]. Additionally, it has been shown that plasma cells and memory B cells in the PB [53], as well as plasma cells with certain specificities in the BM, decrease with age [54]. Considering that, in old age, a suboptimal immune response can be seen in both humans and mice, understanding the intrinsic changes of B cells may offer biological biomarkers useful for measuring the quality of the humoral response [55].

### T cells

One of the leading causes of immunosenescence and aging itself is the involution of the thymus [56]. After birth, the functional parts of the thymus (cortex and medulla) start to be replaced by fat [57,58]. By the age of 45–50, the production of new naive T cells has almost completely ceased. For this reason, in old age, the numbers of naive T cells are reduced in the periphery and in lymphoid organs [30,59–61]. Thus, due to the impaired generation of naive T cells caused by thymic involution, in middle/old age protective immunity mainly depends on the maintenance of memory cells generated in early life after infection, and following vaccinations. In accordance to this, aging is accompanied by increased numbers of effector/memory T cells in the periphery [61,62]. In addition to ‘healthy’ memory T cells, highly differentiated-senescent like T cells accumulate in the elderly. Although both CD4^+^ and CD8^+^ T cells are affected by aging, age-related changes are more evident in CD8^+^ T cells. In the elderly, high frequency of CD8^+^ T cells lack co-stimulatory molecules important for T cell activation and proliferation, such as CD28 [63]. In addition, when becoming late-differentiated, both CD8^+^ and CD4^+^ T cells gain the expression of CD57 [64,65]. CD8^+^CD28^−^ T cells are characterized by short telomere length, reduced antigen-induced proliferation, and enhanced production of pro-inflammatory cytokines [63,66–68].

For these reasons, CD8^+^CD28^−^ T cells have often been considered a population of senescent T cells and are believed to contribute to a low grade age-associated inflammatory background (‘inflammaging’) [69]. The accumulation of this subpopulation of highly differentiated CD8^+^ T cells has also been associated with a reduced immune response to pathogens and vaccines in old age [70]. Similar to the PB, numbers of CD4^+^ and CD8^+^ naive T cells declines while effector memory T cells increase in the BM in old age [23,71,72]. When comparing the frequency of naive and effector/memory CD4^+^ and CD8^+^ T cells in paired PB and BM samples, naive T cells were reduced while effector/memory T cells accumulated in the BM, underlining the importance of BM in the maintenance of immunological memory [23]. In addition, numbers of CD8^+^CD28^−^ T cells increase during aging in the BM [2]. Higher frequency of polyfunctional CD4^+^ and CD8^+^ T cells, T cells which simultaneously produce multiple pro-inflammatory cytokines such as interferon γ (IFNγ) and as tumor necrosis factor (TNF) [73], resides in the BM compared with PB, in both younger and old persons [23].

The function of cytotoxic T cells in BM is not completely understood. However, it is known that these cells play an important role within the BM environment because of their IFNγ production. IFNγ promotes the release of hematopoietic cytokines, including IL-6, from BM mesenchymal cells in the HSC niche, which at low concentrations stimulates hematopoiesis and induces myeloid differentiation [74]. Despite this, the levels of IFNγ in the BM must be well controlled, as it has been shown that osteoclast formation and bone loss are promoted in the presence of this cytokine [75].

In summary, although life-long maintenance of polyfunctional effector/memory T cells and T cell cytotoxicity in the BM seem to be unaffected by age, immunosenescence does take place with advancing age. Indeed, the phenotype of effector/memory T cells that reside in BM niches in old age is very different to the state in younger individuals, in regard to the expression of co-stimulatory molecules (CD28) and cytokine receptors (CD127, CD122) [71].

## Age-related changes in the BM niches: how immunosenescence and age-related inflammation may affect the maintenance of immunological memory in the BM

As the immune system ages, the production of pro-inflammatory molecules such as TNF, and IL- 6 increases in the serum over time [76,77]. This situation has been described as ‘inflammaging’ and it is characterized by a low systemic inflammatory background, which promotes pathophysiological risks throughout the body, contributing to the onset of age-related diseases [69,78]. Chronic exposure to antigens, particularly to persistent viruses, may play an important role in this process, as it repeatedly activate immune cells, which produce high levels of pro-inflammatory molecules; therefore, contributing to ‘inflammaging’. [69,79].

We recently described the effects that aging has on the production of some survival factors for effector/memory T cells and long-lived plasma cells in the human BM [2]. In particular the levels of IL-7, cytokine promoting the survival of memory T cells, were reduced in BM in old age, in accordance with the work performed by Stephan *et al.*, in which BM stromal cells derived from old donors produced less IL-7 than stromal elements derived from young BM [80]. IL-15, preferentially supporting the maintenance of highly differentiated, senescent-like T cells, was increased in old age in the BM [2,23]. Despite this, decreased serum and muscle IL-15 levels were found in old age in mice, suggesting that the age-related accumulation of this cytokine may be organ specific rather than systemic [81]. In addition, plasma cell survival factor APRIL decreased while IL-6 increased during aging in the BM. Chemokine CXCL-12, important in recruiting plasma cells into the niches [10], was not affected by age. Thus, aging may lead to impairments in adaptive immune cell survival in the BM, but the migration of these cells to the niches may not be compromised in old age. It has been hypothesized that accumulation of IL-15 in the BM may be beneficial as it drives the activation and proliferation of effector/memory CD8^+^ T cells in humans and mice [17,23]. Despite this, it is important to consider that both IL-15 and IL-6 are not only involved in the maintenance of adaptive immune cells, but they are also pro-inflammatory cytokines, and therefore, their levels must be kept under control. As these molecule were over-expressed in the aged BM, we hypothesized that inflammation may be responsible, or at least contribute, to age-related impairments in the maintenance of immunological memory in the elderly.

After having described that the levels of both IFNγ and TNF increase in the BM in old age, we also observed that IFNγ in particular could induce the production of IL-15 and IL-6 in myeloid cell types *in vitro* [2]. It has been demonstrated that apoptosis of highly differentiated CD8^+^CD28^−^ T cells could be prevented by IL-15, suggesting that this cytokine may play a role in the survival and the age-related accumulation of CD8^+^CD28^−^ T cells [82]. As we observed the frequency of CD8^+^CD28− T cells increased during aging in the BM, we theorize that IL-15 may strongly support the survival of this subset of highly differentiated T cells [2]. In addition, high levels of IFNγ and TNF were produced by CD8^+^CD28^–^ T cells in the BM; therefore, BM niches involved in the long-term maintenance of memory T cells, appear to progressively become pro-inflammatory in old age, with CD8^+^CD28^−^ T cells supporting this process. Indeed, after their recruitment to the BM, CD8^+^CD28^−^ T cells secrete IFNγ and TNF, which may act on BM cells and promote the production of IL-15 and IL-6, promoting the attraction of new CD8^+^CD28^−^ T cells as a result. Therefore we suggest that a vicious cycle of inflammation may take place in the aged BM, compromising the maintenance of immunological memory as a result.

## The impact of CMV on immunosenescence and the maintenance of immunological memory in the BM

Another factor which may contribute to the age-related dysfunctions in the maintenance of immunological memory in the BM is CMV. CMV is a highly prevalent, persistent β-herpes virus establishing a life-long infection, during which functional changes in the phenotype of T cells take place [83,84]. Within the host, CMV can infect several cell types, including connective tissues, hematopoietic cell types, epithelial and endothelial cells, fibroblasts and smooth muscle cells [85]. Despite this, CMV infection of BM cells represents a rare event [86], and therefore, a direct effect of this virus on the BM environment can generally be excluded.

The immune response to CMV is primarily controlled by cytotoxic CD8^+^ T cells, and a high fraction of the CD8^+^ T cell compartment is required to fight the virus. It is believed that 10% or more of circulating T cells are required to recognize CMV antigens and control the viral proliferation [87]. Even in healthy young people, the virus may not be as innocuous as originally believed. As the body starts fighting against CMV, a very high amount of immune resources are needed in order to keep the viral proliferation under control. This results in consistent stress for the body and leads to several dysfunctions in both, adaptive and innate immunity, thus contributing to immunosenescence. During an acute and chronic CMV infections, T cells undergo several rounds of activation, and therefore, high numbers of CMV-specific effector, and highly differentiated CD8^+^ T cells accumulate throughout the body, persisting for a long time, even when the infection becomes latent [88,89]. Among all the organs, senescent-like CD8^+^ T cells have been shown to accumulate in the BM of CMV^+^ individuals [71]. In seropositive persons, the frequency of CMV-specific terminally differentiated CD8^+^ T cells with reduced levels of CD28, and expressing senescence markers such as killer cell lectin like receptor G1 (KLRG1) dramatically increases in comparison to their seronegative counterparts [71]. This occurs not only in the periphery, but also in the BM; therefore in both the BM and PB a fraction of CMV-specific T cells is unable to react sufficiently against viral antigens due to their terminally differentiated phenotype [90, 91]. In addition, increased interactions between CD8^+^ T cells and IL-15-producing cells have been reported in the presence of CMV [71]. As late differentiated CD8^+^ T cells preferentially respond to IL-15, due to their high expression of CD122 (IL-2/IL-15Rβ) and reduced levels of IL-7Rα, the increased levels of IL-15 [2,71] may support the accumulation of this population during aging and with CMV. In the presence of the virus, the maintenance of ‘healthy’ IL-7Rα^+^CD28^+^ memory T cells is therefore impaired. CMV infection is associated with and may contribute to inflammaging’. Highly differentiated, antigen-experienced CD8^+^CD28^−^ T cells with a pro- inflammatory phenotype accumulate with CMV in the PB and they may also migrate to BM when they circulate [92,93]. CMV infection has also been linked to increased CRP levels in the blood and diseases with an inflammatory component, such as cancer and cardiovascular diseases [94–96]. A negative effect of CMV on the responses to pathogens and vaccines has also been reported. CMV seropositivity triggers a lower antibody (Ab) production after influenza vaccination in young and older adults [97,98]. Low levels of granzyme B activity in response to influenza challenge was reported in CMV^+^ persons [99]. 5 years after booster vaccination against diphtheria, the concentrations of diphtheria-specific Abs are lower in CMV^+^ compared with CMV-individuals [100]. Therefore, as BM plays an important role in maintaining memory T cells and long-lived plasma cells, which guarantee protection against systemic antigens, we can hypothesize that CMV may somehow cause a stress for the BM environment. Indeed, in the presence of the virus, increased frequency of highly differentiated T cells may support BM inflammation, leading to impaired immune responses in the periphery as a result.

## Other factors which limit the maintenance of immunological memory in the BM: oxidative stress, cellular senescence, and competition for space in the BM environment

In addition to inflammation, reactive oxygen species (ROS) may also interfere with the survival of adaptive immune cells in the BM. This hypothesis is supported by the observation that infiltrating leukocytes such as macrophages may constantly produce oxygen radicals, which play an important role in killing pathogens, eventually contributing to structural damages of tissues [78]. Aging itself, as well as proinflammatory molecules, have been shown to regulate ROS levels, contributing to oxidative stress, which represents a typical hallmark of aging [101,102]. *In vitro*, PBMC stimulation with IFNγ leads to increased ROS levels in blood-derived (CD11chi CD14^+^) myeloid cell types [2]. In parallel with the increased levels of IFNγ and TNF in the BM with age, higher ROS levels were found in the pro-inflammatory BM environment of elderly donors. In addition, ROS support the IFNγ-mediated induction of IL-15 and IL-6, as the over-expression of both cytokines was blocked when IFNγ was incubated in the presence of antioxidants N-acetylcysteine (NAC) and vitamin C [2]. Therefore, inflammation may induce ROS, supporting the survival of highly differentiated T cells in the BM as a consequence. The importance of oxidative stress in promoting BM inflammation and inducing the production of IL-15 and IL-6 was described in mice lacking the isoform 1 of antioxidant enzyme superoxide dismutase 1 (SOD-1-/- mice), in which high levels of IFNγ, TNF, IL-15, and IL-6 were observed [2]. In addition, a negative correlation between ROS levels in the BM and diphtheria Ab levels in the plasma was recently observed by our lab [103]. These samples were collected from a large number of healthy donors, and may indicate that ROS somehow impairs the maintenance of long-lived plasma cells in the BM. Further *in vivo* experiments; however, would need to be conducted to confirm these results. These data all provide further evidences about the important role that the BM plays in supporting adaptive immune cells against systemic pathogens.

Age-related inflammation may also be due to cellular senescence [104,105]. Senescent cells, which accumulate with age in many tissues, are thought to drive aging and age-associated diseases through their secretory phenotype, commonly known as senescence-associated secretory phenotype (SASP). Indeed, senescent cells, such as senescent fibroblasts, secrete pro-inflammatory cytokines that modify the tissue microenvironment and alter the function of nearby cells [106]. The elimination of senescent cells in aged mice prevents several age-related pathologies [107,108], and the presence of senescence cells in the BM has shown to be disadvantageous to peripheral antibody concentrations. We also theorize that the age-related changes in the BM may be attributed to a competition for space [103]. This hypothesis follows the observation that, in old age, marrow is dramatically reduced within the bone. Indeed, the percentage of marrow space occupied by hematopoietic cells goes from 40–60% in young adults to 20–40% in elderly persons [4]. We therefore questioned whether physical space limitations exist in the BM marrow niches, with senescent-like effector T cells, which expand in old age, preventing the accumulation of beneficial memory cells. Thus, the niche capacity may be determined by the limited availability of survival factors such as IL-7 and APRIL, production of which is reduced in the aged BM [2], and different cell populations may compete for the same survival factors. When profiling immune cells in the human BM, T cells were seen to increase in the BM with age, while an increase in T cells was correlated with a decrease in B cells and monocytes, suggesting that these populations may potentially be in competition with one another for space. Similar findings were observed for pro-inflammatory T cell populations in the BM, which negatively correlated with B cells. These data suggest that, as highly-differentiated and pro-inflammatory cells accumulate in the BM, less space may be available for other important adaptive immune cells such as B cells and long-lived plasma cells [103].

## Possible interventions to improve the maintenance of immunological memory in the BM

**Figure 2:**
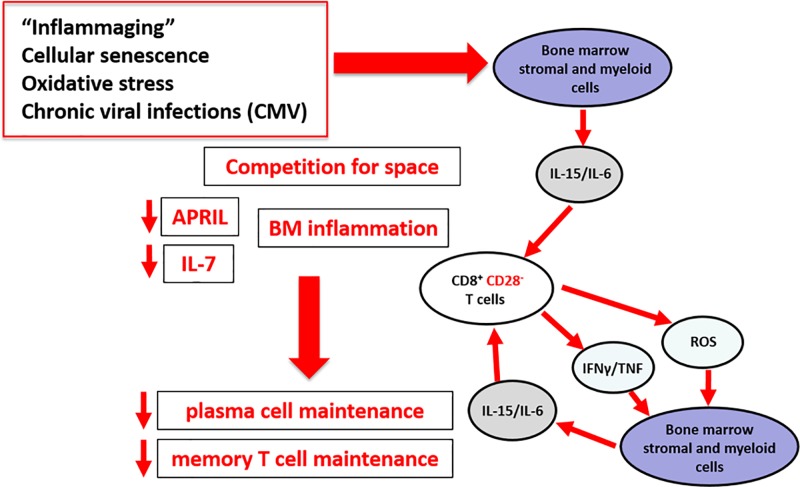
The impact of ‘inflammaging’, cellular senescence, oxidative stress and CMV on the maintenance of immunological memory in the BM

In the elderly, oxidative stress, age-related inflammation (inflammaging), and cellular senescence alter the BM environment, possibly changing the structure of BM niches themselves (Figure 2). In addition, oxygen radicals and pro-inflammatory molecules may support the recruitment of highly differentiated T cells in the BM, which may lead to the vicious ‘ROS-inflammation-cellular senescence’ cycle within the marrow. As the space in the BM is restricted, particularly in old age, infiltrating highly differentiated T cells may occupy space otherwise available for memory T cells and long-lived plasma cells; therefore, excluding them from the survival niches. For this reason, controlling the levels of ROS and pro-inflammatory molecules, for example, by the use of antioxidants, may be beneficial in counteracting the infiltration of late-differentiated T cells in the BM, promoting an optimal maintenance of immunological memory in the BM in the elderly. Pharmacological interventions to reduce oxidative stress, such as lipid peroxidation inhibitors, and anti-inflammatory drugs which specifically target inflammation, may help rejuvenate BM niches [109]. As antioxidants have been shown conflicting results in human trials, the administration of these molecules must be regulated, in order to avoid unwanted side effects.

Despite this, vitamin C and NAC are very safe; therefore offering promising strategies to boost adaptive immunity.

Other possible interventions may be beneficial to combat the impact of aging on BM. In particular, physical activity (cycling) has been shown to increase IL-7 and decrease IL-6 serum levels, improve thymus aging, and reduce Th17 polarization and B regulatory cell frequency [50]. In addition, caloric restriction in mice supports the reduction of IL-6 and TNF levels in the serum [110]. Whether physical exercise and caloric restriction may help counteracting immunosenescence in the BM environment must be investigated in future studies.

## Abbreviations

Ab: antibody
APRIL: a proliferation-inducing ligand
BM: bone marrow
CMV: cytomegalovirus
HSC: hematopoietic stem cell
IFN γ: interferon γ
LLPC: long-lived human plasma cell
MPEC: memory progenitor effector T cell
NAC: N-acetylcysteine
PB: peripheral blood
PBMC: peripheral blood mononuclear cell
ROS: reactive oxygen species
TNF: tumor necrosis factor
VCAM-1: vascular cell adhesion molecule 1
